# Migraine: the seventh disabler

**DOI:** 10.1186/1129-2377-14-1

**Published:** 2013-01-10

**Authors:** Timothy J Steiner, Lars J Stovner, Gretchen L Birbeck

**Affiliations:** 1 Department of Neuroscience, Norwegian University of Science and Technology, Trondheim, Norway; 2 Michigan State University, East Lansing, Michigan, USA; 3 Chikankata Health Services, Mazabuka, Michigan, Zambia

## 

With the agreement of the Editors-in-Chief, this editorial is published simultaneously by *Cephalalgia*, *Headache* and *The Journal of Headache and Pain.*

On 15^th^ December 2012, a special edition of *Lancet* published the principal findings of the *Global Burden of Disease Survey 2010* (GBD2010). Few reports are likely to have more profound meaning for people with headache, or carry greater promise for a better future, than the seven papers (and one in particular [1]) that were presented.

GBD2010 was not the first such survey to be conducted, nor the first to give some recognition to the burden of migraine. The *Global Burden of Disease Survey 2000* (GBD2000), conducted 12 years ago by the World Health Organization (WHO), listed migraine as the 19^th^ cause of disability in the world, responsible for 1.4% of all years of life lost to disability (YLDs) [2]. This finding has been cited repeatedly ever since; it has fuelled attempts to generate political acceptance of headache as a public-health priority [3], and given credibility to calls for greater investment in headache care and research. It pushed headache into WHO’s field of view, and became an essential part of the platform on which the Global Campaign against Headache has since been built [3-5].

In spite of all this, GBD2000 considerably underreported the disability that migraine imposed on people throughout the world, and gave a very poor account of headache disorders collectively. The evidence was not there. For more than half the world’s population, estimates for migraine were based on very little: data of acceptable quality were not in existence for China, India and most other countries in South East Asia, most of Africa, all of the Eastern Mediterranean and all of eastern Europe [6]. Headache disorders other than migraine did not feature in GBD2000 at all; for these disorders, at that time, dependable evidence was lacking everywhere.

Filling this evidence gap has been a priority of the Global Campaign in its first years [7]. As a result, GBD2010 has been much better informed and built on much sounder foundations than its predecessor (we return to this point later). GBD2010 was not a simple update of GBD2000, but a complete rerun: an entirely new world survey. Working with many partners, the Global Campaign against Headache being one, it took from the world literature all the epidemiological evidence pertaining to burdensome diseases, assessed it for quality and derived from it, for each of 21 world regions, best age-related estimates of prevalence. Like GBD2000, it measured burden in disability-adjusted life years (DALYs), separated into the two components of YLDs and years of life lost to early mortality (YLLs); for headache, only the former are relevant. New disability weights (DWs) were assigned to each disease: lay descriptions of the various health states that were predictable sequelae of each disease were fed into a web-based worldwide consultation, which conducted an iterative series of comparisons, one health state with another.

For migraine and tension-type headache (TTH), descriptions were agreed of average cases and three health states of each: ictal (during attacks), interictal (between attacks), and the health state associated with medication-overuse headache (MOH), which was considered as a potential complication of either. Information from published studies on frequency and duration of migraine or TTH episodes was pooled in order to estimate the average proportions of time (pT) spent in the ictal as opposed to interictal state. MOH was assumed to be continuous (pT=1) when present. YLDs for each of these states were then derived as products of prevalence, pT and DW, and for each disease as the sum of YLDs for each health state. Data were included from 84 studies of migraine in 43 countries in 16 of the 21 world regions, and from 45 studies of TTH in 34 countries in 13 world regions.

TTH (estimated global prevalence 20.1%) and migraine (14.7%) ranked respectively as second and third most common diseases in the world (behind dental caries) in both males and females. For migraine, the estimated proportion of time spent in the ictal state was 5.3%, and the DW assigned to migraine episodes was 0.433 (43.3% disability). On the basis of ictal disability alone, migraine was ranked seventh highest among specific causes of disability globally (responsible for 2.9% of all YLDs), and in the top ten causes of disability in 14 of the 21 world regions, showing little evidence of a gradient falling from west to east or of being a disorder preferentially of rich countries. Migraine was, by a wide margin, the leading cause of disability among neurological disorders, accounting for over half of all YLDs attributed to these. For TTH, the estimated proportion of time spent with headache was 2.4%, and the DW assigned to headache episodes was 0.040 (4% disability). TTH accounted for only 0.23% of all YLDs, much less than predicted [6], which undoubtedly was because of the very low DW accorded to the ictal state.

Regrettably, GBD2010 is still an incomplete account of the global burden of headache, and it continues to underestimate the disability arising from headache disorders. TTH got in, but MOH, which would probably have added much more substantially to the total YLDs, was excluded late in the survey for reasons not made clear and despite the evidence submitted in support of it. Also at a late-stage, the inclusion of interictal disability was considered inconsistent with measurements made of other chronic episodic conditions, which penalized migraine more than TTH. Even so, this very high-profile survey of the world’s causes of ill health better recognizes headache than anything before, and this is a big step forward.

We might be satisfied by this; but rather we should be appalled. GBD measures disease burden as it is – alleviated by whatever treatments are made available. Headache disorders are among the top ten causes of disability because they are common and disabling; that is clear. Headache is one of the most frequent medical complaints: almost everybody has experienced it, at least 10% of adults everywhere are sometimes disabled by it, and up to 3% live with it on more days than not [6]. But for what conceivable reason do headache disorders remain among these ignominious top ten when they are largely treatable? Another recent global survey, conducted collaboratively by WHO and *Lifting The Burden*, described “worldwide neglect of major causes of public ill-health, and the inadequacies of responses to them in countries throughout the world” [8]. It drew attention to the very large numbers of people disabled by headache who do not receive effective health care. The barriers responsible for this might vary throughout the world, but poor awareness of headache in a context of limited resources generally – and in health care in particular – was constantly among them [8]. The consequences are inevitable: illness that can be relieved is not, and heavy burdens, both individual and societal [9], persist when they can be mitigated. The findings of GBD2010 sadly reflect this. GBD2010 sends out a clarion call, conveying a message of which governments need to take note [3]. Experience suggests this call will need constantly to be re-echoed, but the opportunity to use GBD2010 – for a better future for people with headache – must not be missed.

## Competing interest

The authors served on the Neurologic Disorders Expert Group in Headache for the Global Burden of Disease 2010 Study (funded by the Bill & Melinda Gates Foundation), and are directors and trustees of *Lifting The Burden*, which conducts the Global Campaign against Headache in official relations with WHO. TJS is honorary Global Campaign Director.
